# Performance Validation of Fabricated Nanomaterial-Based Biosensor for Matrix Metalloproteinase-8 Protein Detection

**DOI:** 10.1055/s-0045-1809182

**Published:** 2025-05-21

**Authors:** Pimsunee Lowpradit, Rapiphun Janmanee, Kallapat Tansriratanawong

**Affiliations:** 1 Department of Oral Medicine and Periodontology, Faculty of Dentistry, Mahidol University, Bangkok, Thailand; 2 Department of Chemistry, Faculty of Science and Technology, Pibulsongkram Rajabhat University, Phitsanulok, Thailand

**Keywords:** biosensor, nanomaterials, matrix metalloproteinase-8, periodontitis, point-of-care testing

## Abstract

**Objective:**

Matrix metalloproteinase-8 (MMP-8) is a crucial collagenase enzyme that primarily degrades type I collagen and extracellular glycoproteins, playing a significant role in the pathological processes of periodontal disease. It can serve as a biomarker for early detection and screening of the disease through advanced biosensor technology. The aim of this study was to fabricate and validate the performance of a nanomaterial-based biosensor for detecting MMP-8 protein.

**Materials and Methods:**

The screen-printed gold electrode was modified with a thin film of 11-mercaptoundecanoic acid using the self-assembled monolayer technique. A biosensor was then created with N-hydroxysuccinimide and 1-ethyl-3-(3-dimethylaminopropyl) carbodiimide to form ester bonds, followed by the immobilization of the antibody MMP-8 and blocking of nonspecific binding. The performance characteristics of a biosensor for detecting MMP-8 concentrations, ranging from 1 to 50 ng/mL, were evaluated using electrochemical techniques with data analysis performed using the NOVA software. Enzyme-linked immunosorbent assay (ELISA) was used as a control.

**Statistical Analysis:**

The results were expressed as mean values ± standard deviation. The coefficient of determination ( *R* ^2^ ) was calculated based on the obtained calibration curve.

**Results:**

Electrochemical measurements revealed that the peak current after modifying the thin film on the electrode was lower than the bare electrode. Characterization of biosensors showed an increase in response compared to the previous step. Differential pulse voltammetry measurements indicated that the peak current for MMP-8 concentrations ranging from 1 to 50 ng/mL increased proportionally with concentration. The biosensor demonstrated high sensitivity, with a correlation coefficient of *R* ^2^ = 0.953 when compared to ELISA ( *R* ^2^ = 1).

**Discussion and Conclusion:**

A biosensor utilizing nanomaterials has been successfully fabricated for the detection of the MMP-8 protein with high sensitivity. Subsequent research should prioritize the evaluation of its performance in clinical patients alongside an assessment of its specificity and stability. The objective was to advance this biosensor as a reliable diagnostic tool for the screening of periodontitis.

## Introduction

Periodontal disease is a chronic inflammatory disease that damages the support structures of the teeth. The disease manifests through various inflammatory reactions, typically caused by an imbalance between periodontal pathogens and the host's immune response, a condition known as dysbiosis. 1 2 3 4 The progression of periodontal disease frequently occurs asymptomatically, remaining undetected until advanced stages are reached. At this stage, patients typically experience a considerable reduction in clinical attachment level (CAL), significantly increasing their risk of tooth loss. Therefore, early detection of periodontal disease is crucial for effective treatment. Timely intervention can impede the condition's progression.

The pathological processes of periodontal disease trigger the release of several inflammatory cytokines, which damage the periodontium, including interleukin-1 beta (IL-1β), tumor necrosis factor alpha (TNF-α), and matrix metalloproteinases (MMPs). 5 MMP-8, a specific type of MMP, is involved in the early stages of inflammation and is present at higher levels in patients with periodontal disease compared to healthy individuals. 6 7 Several studies have highlighted the significant role of MMP-8, demonstrating increased levels in gingival tissue, gingival crevicular fluid (GCF), and saliva in individuals with chronic periodontitis. MMP-8 is recognized as one of the biomarkers for periodontitis screening. 6 8

A complete full-mouth periodontal examination depends on the clinician's expertise to gather significant data, such as CAL, probing depth, bleeding on probing, furcation involvement, and tooth mobility for an accurate diagnosis. However, documenting all readings from six sites per tooth is labor-intensive. Thus, screening methods for periodontal disease offers an efficient alternative for timely patient intervention.

Several methods are available for screening periodontal disease, including questionnaires, periodontal screening record, and self-reported data. 9 10 11 12 Goulão et al described a periodontal diagnostic model for self-reported oral health, showing high specificity for gingival bleeding (88%). In contrast, bad breath and unpleasant taste had lower specificity (39–72%). The accuracy, indicated by the area under the curve (AUC), was modest (0.52–0.60). 12 Although these methods can improve assessment efficiency, their subjective nature and reliance on self-reporting limit their ability to reflect the relationship with periodontal disease fully. 9 10 Point-of-care (POC) testing is increasingly used for detection, prognosis, and diagnosis due to its ease of use, speed, low cost, environmental sustainability, and reliability. 13 POC testing for periodontal disease diagnosis uses microbiological and immunological biomarkers. However, these tests often suffer from low sensitivity and specificity, complex methodologies, and time consumption. 14 Therefore, developing methods that offer high sensitivity and specificity with faster results is crucial for effective periodontal screening.

POC tests for MMP-8 in enzyme-linked immunosorbent assay (ELISA) show an average sensitivity of 87% and specificity of 60%, with sensitivity in severe periodontitis patients reaching up to 93%. 15 However, POC testing varies widely, with sensitivity ranging from 70 to 90% and specificity from 50 to 70%. 16 Therefore, to enhance biosensor performance, novel techniques have been developed, including antibody-based biosensors, 17 cell-secreted on-chip dual-sensing devices, 18 and nanomaterial-based biosensors. 19 20

Nanomaterials are solid particles sized between 1 and 100 nanometers (nm). 21 The key property of nanomaterials is their high reactive surface area, and their small particle size enables high-parallel and highly efficient capture of specific target molecules. 22 23 In periodontal disease, nanomaterials are components of electrochemical biosensors based on electrochemical principles, which can enhance sensitivity and specificity. 24 25 Recently, Tortolini et al pioneered the establishment of a voltammetric biosensor using nanoparticles to detect MMP-8. This biosensor demonstrated good performance, with a linear detection range of 2.5 to 300 ng/mL, a limit of detection (LOD) value of 1.0 ± 0.1 ng/mL, and a sensitivity of 0.05 µA.mL/ng.cm ^2^ . However, each biosensor was characterized using gold nanospheres on an electrode, which involved Raman spectroscopy measurements. This process complicated the biosensor preparation, making it time-consuming and requiring calibration. Additionally, the results indicated relative standard deviation (SD) values ranging from 4.7 to 12.4% when compared to the ELISA assay. 20

Therefore, this research aimed to fabricate and validate the performance of a nanomaterial-based biosensor for detecting MMP-8 protein. The fabricated biosensors are expected to provide preliminary information that could lead to the development of effective, rapid, and inexpensive POC testing.

## Materials and Methods

### Materials and Apparatus

MMP-8 and MMP-8 antibodies (anti-MMP-8) were purchased from Sigma-Aldrich (Missouri, United States). All other chemicals were analytical grade with the highest purity: potassium ferricyanide (III) (K _3_ [Fe(CN) _6_ ]), potassium ferrocyanide (II) (K _4_ [Fe(CN) _6_ ]), ethanol, 11-mercaptoundecanoic acid (11-MUA), N-hydroxysuccinimide (NHS), 1-ethyl-3-(3-dimethylaminopropyl) carbodiimide (EDC), and ethanolamine hydrochloride (EA-HCl) were purchased from Sigma-Aldrich.

All solutions, 5 mM solution of ferrocyanide/ferricyanide [Fe (CN) _6_ ] ^3- /4-^ 1:1 ratio, 0.4 M EDC, 0.1 M NHS, and 0.2 M EA-HCl, were prepared in phosphate-buffered saline (PBS), pH 7.4. PBS was used as the buffer in all experiments and prepared from PBS tablets in deionized water. All solutions were stored under 4°C before performing the experiment.

**Fig. 1:**
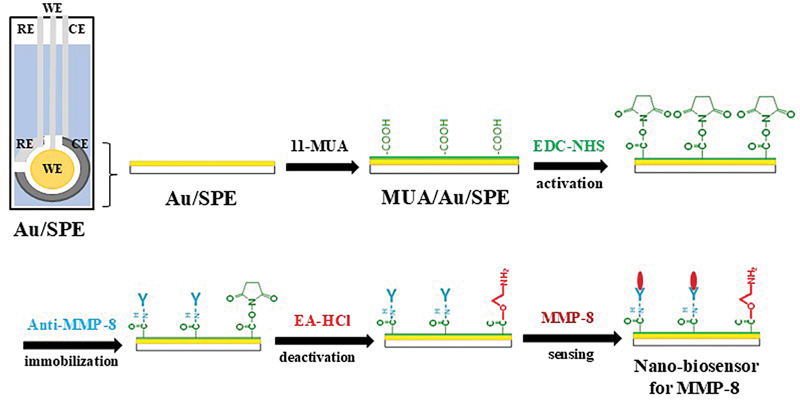
Fabrication of biosensors: The experimental setup involved three electrodes: a reference electrode (RE) made of silver (Ag), a counter electrode (CE) made of platinum (Pt), and a working electrode (WE) made of gold (Au). The bare Au screen-printed electrode (Au/SPE) was modified using 11-mercaptoundecanoic acid (MUA) to create a MUA/Au/SPE configuration. Next, the antibody was immobilized on the surface using EDC-NHS for 30 minutes, followed by the addition of 40 μg/mL of anti-MMP-8 and allowing it to incubate for another 20 minutes. Lastly, a 0.2 M EA-HCl buffer solution was applied for 15 minutes to block any nonspecific binding. Au/SPE, screen-printed gold electrodes; MUA/Au/SPE, modified-Au/SPE by 11-mercaptoundecanoic acid; EDC/NHS, N-ethyl-N'-(3-(dimethylamino)propyl) carbodiimide/N-hydroxysuccinimide; anti-MMP-8, matrix metalloproteinase-8 antibody; EA-HCl, ethanolamine hydrochloride.

A screen-printed gold electrode (Au/SPE) (250AT, diameter 4 mm, Metrohm, Switzerland) consisted of three electrodes: the reference electrode (RE) was silver (Ag), the counter electrode (CE) was platinum (Pt), and the working electrode (WE) was gold (Au), which are coated with high-temperature process ink printed on a solid substrate ( Fig. 1 ).

Electrochemical measurements, including cyclic voltammetry (CV) and differential pulse voltammetry (DPV), were performed on a PC-controlled AUTOLAB PGSTAT204 potentiostat-galvanostat (Metrohm Autolab B.V., Netherlands). The CV and DPV measurements were recorded by scanning from –0.3 to 0.6 V, amplitude 20 mV, and step potential 5 mV. Baseline corrections were done for all data using the NOVA software.

Scan rate, a term used to measure the speed of voltage changes over time, is defined in electrochemistry as the rate at which the potential is varied during electrochemical measurements. It is typically expressed in volts per second (V/s). This parameter is crucial because it affects the current response and the overall shape of the voltammetric curve, influencing both the resolution and sensitivity of the measurement. A higher scan rate can lead to faster experiments but may also reduce peak heights and alter the redox reaction.

In all steps of the process, a 5-mM solution of [Fe(CN) _6_ ] ^3−^ / ^4−^ was used to enhance the visibility of the higher peaks. Characterization of the modification step of biosensor surface was performed using CV and DPV measurements.

### Fabrication of Au/SPE-Modified Electrode

A 5-mM solution of 11-MUA in ethanol was prepared and allowed to react for 24 hours to fabricate a thin film-based composite nanomaterial on Au/SPE by the self-assembled monolayer method. The morphology of the thin film-based composite nanomaterial was characterized by analyzing the electrochemical behavior of the modified-Au/SPE (MUA/Au/SPE) in PBS solution (pH 7.4), which was monitored by CV and DPV measurements ( Fig. 1 ).

### Construction of Biosensors

The MUA/Au/SPE was rinsed with ethanol to remove any unbound molecules. According to a procedure reported by Tortolini et al, 20 the antibody was immobilized onto the surface using a 1:1 aqueous mixture of 0.4 M EDC and 0.1 M NHS, which was applied to the MUA/Au/SPE for 30 minutes to activate the carboxyl groups on the thin-film electrode surface, forming N-hydroxysuccinimide esters. The electrode was rinsed with PBS solution, and then 40 μg/mL of anti-MMP-8 was added for 20 minutes. The anti-MMP-8 was immobilized onto the activated MUA/Au/SPE surface through the NHS ester group. After rinsing with PBS, a 0.2-M EA-HCl buffer solution was applied for 15 minutes to block any nonspecific binding, and the remaining unreacted NHS esters were deactivated before MMP-8 detection. The electrode surface was washed with PBS buffer solution at the end of each fabrication step. The resulting nanomaterial-based biosensor was used for the detection of various concentrations (range 1–50 ng/mL) of MMP-8 ( Fig. 1 ).

DPV technique was used to evaluate the performance of the composite nanomaterial-based biosensor for the detection of MMP-8. Electrochemical experiments were conducted at a constant potential with MMP-8 stock to record the amperometry responses, allowing for the determination of MMP-8 concentrations.

### ELISA

ELISA is considered the gold standard for detecting a wide range of diseases and is commonly used as a clinical diagnostic tool. ELISA is also used as a reference for plotting a standard curve based on serial dilutions. The SimpleStep ELISA kit for human MMP-8 (Abcam, Cambridge, United Kingdom) was used as a positive control and standardized for comparison with the fabricated nanomaterial-based biosensors. MMP-8 concentrations were diluted in the range of 0 to 5 ng/mL, starting from 5 ng/mL, and serially diluted to 2.500, 1.250, 0.625, 0.313, 0.156, 0.078, and 0 ng/mL, according to the manufacturer's instructions. Then, 50 µL of MMP-8 was added to each well of the 96-well plate, followed by 50 µL of the antibody cocktail. The plate was incubated for 1 hour at room temperature on a plate shaker set to 400 revolutions per minute (rpm). All wells were washed with wash buffer, and 100 µL of 3,3',5,5'-tetramethylbenzidine development solution was added, followed by incubation for 10 minutes in the dark, with the plate shaker set to 400 rpm. Finally, 100 µL of stop solution was added, the plate was shaken for 1 minute, and the results were analyzed using a microplate spectrophotometer (Santa Clara, California, United States) with an optical density (OD) reader set to 450 nm.

### Statistical and Data Analysis

This study was a descriptive analysis. The fabricated nanomaterial-based biosensor was evaluated using electrochemical measurements, including CV and DPV, and data analysis was performed using the NOVA software. The results were expressed as mean values ± SD. Based on the obtained calibration curve, the coefficient of determination ( *R* ^2^ ) was calculated. Additionally, various concentrations of MMP-8 were used to calculate the LOD and limit of quantification (LOQ) from the peak current using the following equations:

LOD = (3 * *S* )/ *m*

LOQ = (10 * *S* )/ *m*

where “ *S* ” is the SD of the peak currents of the blank and “ *m* ” is the slope of the calibration plot.

## Results

### Characterization of Nanomaterials-Modified Electrode

#### Fabrication of SPE-Modified Electrode

**Fig. 2:**
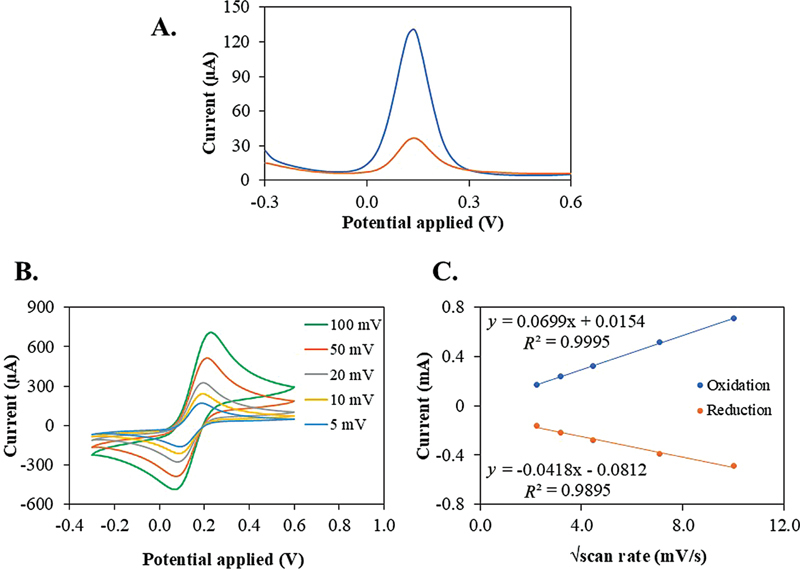
Characterization of the nanomaterials-modified electrode: ( **A** ) DPV voltammograms of bare Au/SPE (blue) and MUA/Au/SPE (orange) at potential range of –0.3 to 0.6 mV. ( **B** ) CV of MUA/Au/SPE-modified electrode at different scan rates and ( **C** ) a linear relationship between the 5 peak currents at different scan rates (5, 10, 20, 50 and 100 mV/s) and the √scan rate. CV, cyclic voltammetry; DPV, differential pulse voltammetry; Au/SPE, screen-printed gold electrodes; MUA/Au/SPE, modified-Au/SPE by 11mercaptoundecanoic acid.

The nanomaterials-modified electrodes used in this study were characterized through voltammetry analysis, specifically employing DPV methods. Fig. 2A shows the peak currents obtained from DPV measurements following the electrochemical fabrication of the MUA/Au/SPE composite thin film. The results indicate that the peak currents for the MUA/Au/SPE composite (represented by the orange wave) are lower than those of the bare Au/SPE (represented by the blue wave).

#### Electrochemical Characterization of MUA/Au/SPE-Modified Electrode

The electrochemical characteristics were illustrated through the redox reaction, which consists of oxidative reactions (positive values) and reductive reactions (negative values) represented on the vertical ( *Y* ) axis. Fig. 2B displays the CV measurements of the MUA/Au/SPE composite thin film at various scan rates of 5, 10, 20, 50, and 100 mV/s. As the scan rate was increased, there was a corresponding rise in the currents of the redox peaks.

A linear relationship was identified between the five peak currents measured at varying scan rates (5, 10, 20, 50, and 100 mV/s) and the square root of the scan rate (√scan rate). This relationship demonstrated a strong correlation, evidenced by an *R* ^2^ value of 0.989 and 0.999, as depicted in Fig. 2C , indicating that the mass and electron transfer occurs at the electrode surface.

#### Construction of Immunosensor

The fabricated nanomaterial-based biosensor was modified sequentially with EDC-NHS, anti-MMP-8, and EA-HCl, as described in the Methods section. The peak currents were obtained from DPV measurements. The results demonstrate that after the MUA/Au/SPE modification, the peak currents gradually slightly increased.

### Validation of MMP-8 Detection (Sensitivity)

**Fig. 3:**
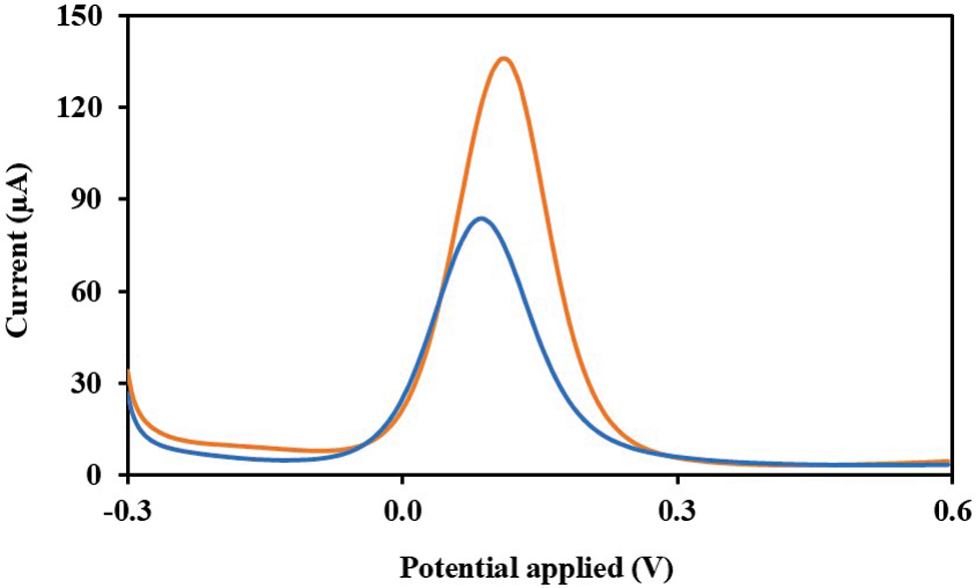
Validation of MMP-8 detection: DPV of the detection of 100 ng/mL MMP-8 concentration (orange line) produced a higher peak current compared to the EA-HCl/anti-MMP-8/EDC/NHS/MUA/Au/SPE electrode configuration (blue line) at potential range of –0.3 to 0.6 mV. DPV, differential pulse voltammetry; MMP-8, matrix metalloproteinase-8; EA-HCl/anti-MMP-8/EDC/NHS/MUA/Au/SPE, the electrode surface at the end of each fabrication step.

The biosensor, prepared and ready for use, was tested with various concentrations of MMP-8. Fig. 3 shows the results demonstrating that MMP-8 concentration produced a higher peak current compared to the EA-HCl/anti-MMP-8/EDC-NHS/MUA/Au/SPE electrode configuration.

**Fig. 4:**
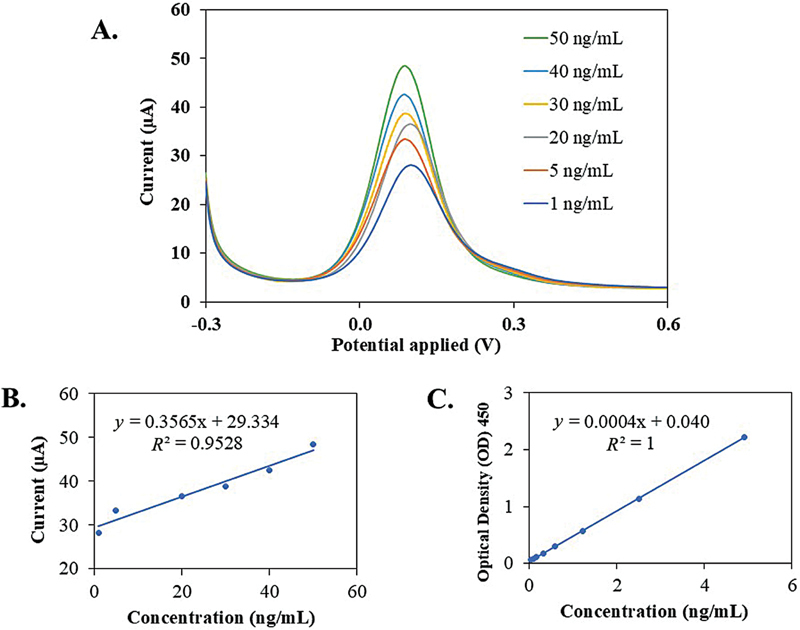
Validation of MMP-8 detection: ( **A** ) DPV voltammograms of the detection of MMP-8 at a concentration range of 1 to 50 ng/mL. ( **B** ) A linear relationship between 6 peak currents of (A), with *R* ^2^ = 0.953. ( **C** ) A standard curve of enzyme-linked immunosorbent assay (ELISA) for MMP-8 concentrations ranges from 0 to 5 ng/mL, with OD at 450 nm, with *R* ^2^ = 1. DPV, differential pulse voltammetry; MMP-8, matrix metalloproteinase-8; *R* ^2^ , correlation coefficient; OD, optical density.

The biosensor was validated by testing various concentrations of MMP-8 in the range of 1 to 50 ng/mL, when the peak current (µA) was plotted against the average time of 0 to 5 minutes from the DPV measurements. Fig. 4A shows the current response for various concentrations of MMP-8, ranging from 1 to 50 ng/mL, within an applied potential range of –0.3 to 0.6 V. The peak current increased in a dose-dependent manner as the MMP-8 concentration increased from 1 to 50 ng/mL.

A linear relationship between peak current and different concentrations at 1, 5, 20, 30, 40, and 50 ng/mL was observed in Fig. 4B , with a correlation coefficient ( *R* ^2^ ) of 0.953. The calculated values for LOD and LOQ were 0.960 and 3.201 ng/mL, respectively. When compared to the ELISA assay as a positive control, the standard curve for various concentrations of MMP-8 (0–5 ng/mL) was obtained, with absorbance measured at 450 nm ( Fig. 4C ). The linear relationship demonstrates a positive correlation between concentration and OD value, with an *R* ^2^ value of 1.

**Fig. 5:**
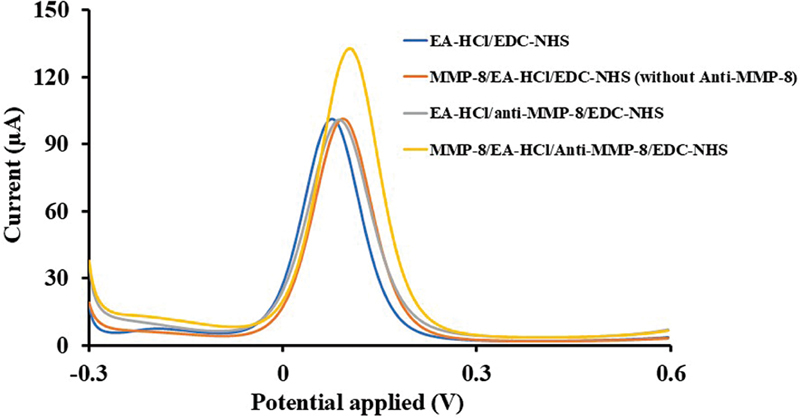
Selectivity of modified biosensor: DPV for detecting MMP-8 based on the modified biosensor with and without antibodies. DPV of the detection of 100 ng/mL MMP-8 concentration (yellow line) produced a higher peak current compared to the EA-HCl/anti-MMP-8/EDC-NHS/MUA/Au/SPE electrode configuration (gray line), 100 ng/mL MMP-8/EA-HCl/EDC-NHS/MUA/Au/SPE electrode configuration (orange line), and EA-HCl/EDC-NHS/MUA/Au/SPE electrode configuration (blue line). DPV, differential pulse voltammetry; MMP-8, matrix metalloproteinase-8; EA-HCl/anti-MMP-8/EDC/NHS/MUA/Au/SPE, the electrode surface at the end of each fabrication step; MMP-8/EA-HCl/EDC-NHS/MUA/Au/SPE, the electrode surface with MMP-8 detection but without MMP-8 antibody.

The evaluation of the selectivity of the modified biosensor is presented in Fig. 5 . The figure shows the DPV response for detecting MMP-8 based on the modified biosensor, both with and without antibodies. The DPV response of the modified biosensor without antibodies exhibited no significant change in current, whereas the proposed electrochemical biosensor displayed a high current response, indicating remarkable selectivity for MMP-8 detection.

## Discussion

This study successfully fabricated the validation of a gold nanomaterial-based biosensor for MMP-8 detection, representing a high sensitivity performance ( *R* ^2^ = 0.953) within a concentration range of 1 to 50 ng/mL. The electrode characteristics revealed a lower peak current for MUA/Au/SPE after modification with 11-MUA compared to the bare Au/SPE. This change confirms that the -COOH groups of 11-MUA at the thin-film interface enhance the binding of biomolecules; however, this may block electron transfer as a side effect. The detection mechanism of MMP-8 is based on the MUA/Au/SPE configuration. Initially, the -COOH groups of 11-MUA on the MUA/Au/SPE surface were activated using an aqueous mixture of EDC/NHS, which formed NHS esters. Subsequently, the anti-MMP-8 was immobilized onto the activated MUA/Au/SPE surface through the NHS ester group. To prevent nonspecific binding, the remaining unreacted NHS esters were deactivated using an EA–HCl buffer solution before MMP-8 detection. Upon MMP-8 immobilization, amide bonds were formed between the NHS ester group on the activated electrode surface and amine groups of MMP-8. This specific interaction reduces nonspecific adsorption, which enhances signal-to-noise ratio of MMP-8 and contributes to high sensitivity.

Biosensor performance demonstrates a similar trend to that of a previous study. 20 Tortolini et al demonstrated the effectiveness of a novel voltammetric biosensor for detecting salivary MMP-8, achieving high sensitivity ( *R* ^2^ = 0.986) and specificity (selectively targeting MMP-8 while excluding MMP-2, MMP-9, and nonspecific analytes). However, the characteristics of gold nanosphere electrodes can differ significantly due to the custom synthesis process, which requires calibration and can be time-consuming for characterization. In contrast, the electrodes used in our study were calibrated by the manufacturer and screen-printed directly onto the substrate. This approach reduces errors associated with electrode construction, indicating that our study offers greater accuracy.

Recently, nanoparticle-based immunochemical biosensors have been widely used in the medical and dental fields. 26 These nanoparticles for immunosensing materials are categorized into metallic nanoparticles (gold and silver nanoparticles), magnetic nanoparticles, carbon-based nanomaterials (such as carbon nanotubes and graphene), and luminescent nanocrystals (quantum dots and photon-upconverting nanoparticles). 22 27 Gold nanoparticles possess several advantageous properties, including simple preparation, convenient fabrication methods, good biocompatibility, and high chemical stability. 28 Consequently, recent research has employed gold nanoparticles for the coating of electrodes aimed at biomarker detection. 29 30 31 32

A systematic review and meta-analysis of salivary MMP-8 as a biomarker for periodontitis revealed a variety of detection methods based on antigen-antibody interactions, including ELISA, Luminex (immunoassay), and immunofluorometric assay (IFMA). However, the concentration range of MMP-8 detection showed significant variation, with reported values ranging from 2.95 to 888.60 ng/mL, indicating heterogeneity in the results. 33 Notably, some studies have reported that the MMP-8 levels in cases of incipient periodontitis, with the minimum detectable level in periodontal disease, showed median concentrations of 22.90 ng/mL in rinse samples 16 and 28.73 ng/mL in GCF samples. 34 In our study, a concentration range of 1 to 50 ng/mL for MMP-8 was selected for the purpose of early detection and screening of periodontitis, which yielded high sensitivity. Furthermore, the low LOD value obtained from our study was 0.960 ng/mL, which is comparable to those reported in previous studies: 1.76 ng/mL 19 and 1.00 ng/mL 20 ( Table 1 ). Therefore, our biosensor has the ability to detect MMP-8, even at low concentrations.

**Table 1:** Analytical performances of different biosensors for MMP-8 detection

Sensing platform	Technique	Linear range (ng/mL)	LOD (ng/mL)	Ref.
Point-of-care immunoflow device (POCID)	Lateral flow chromatography (LFC)	−	−	16
Specific-antibody biochip	Surface acoustic wave (SAW)	0–1000	62.5	17
Optical fiber-based	Surface plasmon resonance (SPR)	4.4–176	1.76	19
MUA/AuNPs/GPH/SPE	DPV	2.5–300	1.0 ± 0.1	20
Periosafe	Lateral flow immunoassay	20–50	25	34
G-UCNPs-LFIS	Disk-like lateral flow immunoassay strip	0–400	10	36
MUA/Au/SPE	DPV	1–50	0.960	This study

Abbreviations: DPV, differential pulse voltammetry; LOD, limit of detection; MMP-8, metalloproteinase-8; Ref., references.

The qualitative detection of the lateral flow immunoassay (Periosafe) has become a standard method used commercially for screening periodontitis. This method can yield both positive results (indicating low-risk or high-risk) and negative results. 6 35 Additionally, He et al developed a nanomaterial-based lateral flow immunoassay using three biomarker fluorescence probes: MMP-8, IL-1β, and TNF-α. This technique can classify patients into categories of healthy, mild periodontitis, and severe periodontitis. However, it does not provide quantitative data despite a high correlation index for the three biomarkers ( *R* ^2^ = 0.976–0.995). 36 Therefore, to improve the detection method for MMP-8 and achieve quantitative results, it is suggested that an antigen-antibody-based biosensor be incorporated to enhance performance. Taylor et al developed a prototype antibody-based biosensor using biochip technology that incorporates surface acoustic wave (SAW) principles. The performance of this biosensor was indicated by an AUC of 0.89, which is slightly lower than that of ELISA at 0.93. However, the SAW biosensor presents some challenges for chairside use due to its laboratory-based prototype design and the time-consuming nature of the testing process. 17

MMP-8 is an inflammatory enzyme in the collagenase group, with a molecular weight of approximately 75 to 80 kDa. 37 The specific antigen-antibody interaction between the anti-MMP-8 antibodies and the MMP-8 makes it an ideal candidate for use in biosensor detection. Larger MMP-8 molecules with multiple epitopes provide more binding sites for antibodies, which could enhance the binding efficiency and sensitivity of the biosensor. Moreover, nanomaterial-based biosensors can be utilized to detect periodontal pathogens, 38 such as *Porphyromonas gingivalis* . 39 40 However, this periodontal pathogen is a Gram-negative bacterium, and it is sensitive to the preparation process. Furthermore, relying on a single species as a predictor of disease progression may not be sufficient due to the complex nature of periodontal disease, which is often associated with dysbiosis—an imbalance in microbial communities. 40

The first limitation of this study is that the concentration of MMP-8 in individuals diagnosed with periodontitis exhibits substantial variability, with values ranging from 2.95 to 888.60 ng/mL. In our study, we specifically focused on concentrations between 1 and 50 ng/mL, with an emphasis on early detection and screening for periodontitis. Therefore, further research is warranted to confirm the presence of higher concentrations up to 50 ng/mL and to develop methodologies for distinguishing between different stages of the disease at varying concentration levels. Second, the stability of the nanomaterial-based biosensor, Tortolini et al demonstrated that their biosensor maintained about 90% of the DPV response after 2 weeks. However, it is advisable to utilize the biosensor as soon as possible upon noticing any degradation in the antigen-antibody reaction. Additionally, investigations involving periodontitis patients should consider modifying or fabricating a combination of biomarkers into a single diagnostic tool and utilizing GCF and saliva as diagnostic tools for these patients. Future research should focus on evaluating its performance in clinical patients, as well as assessing its specificity and stability, to further develop this biosensor into a reliable diagnostic tool for screening periodontitis.

## Conclusion

This study has successfully established a nanoparticle-based biosensor for the detection of MMP-8, demonstrating high sensitivity. Further investigations are recommended to evaluate the performance of the biosensor in clinical settings and assess its specificity and stability. The goal is to establish this biosensor as a reliable diagnostic tool for periodontitis.
